# Biomarkers of *in vivo* platelet activation in thoroughbreds during their first long-term training

**DOI:** 10.3389/fvets.2024.1395423

**Published:** 2024-05-20

**Authors:** Arianna Miglio, Emanuela Falcinelli, Anna Maria Mezzasoma, Sara Busechian, Fabrizio Rueca, Paolo Gresele, Maria Teresa Antognoni

**Affiliations:** ^1^Department of Veterinary Medicine, Perugia University, Perugia, Italy; ^2^Department of Medicine and Surgery, Section of Internal and Cardiovascular Medicine, Perugia University, Perugia, Italy

**Keywords:** soluble P-selectin, platelet-derived extracellular vesicles, equine, exercise, platelet

## Abstract

Physical exercise has an activating effect on platelet function that differs between trained and untrained subjects, depending on the type of exercise and training status. In humans, soluble P-selectin (sP-sel) and platelet-derived extracellular vesicles (PEVs) are considered reliable markers of *in vivo* platelet activation during exercise. In untrained humans, they increase after transient physical exercise, whereas long-term training induces a decrease in their resting levels due to an improved ability to adapt to hemodynamic changes. The aim of this study was to assess whether circulating levels of sP-sel and PEVs may be useful markers to explore *in vivo* platelet function in never-trained Thoroughbreds during their first 4 months of incremental training. A total of 29 clinically healthy, untrained Thoroughbreds (17 males and 12 females) were enrolled. All horses were trained with the same training schedule (90 days). Blood samples were collected on the day the training program began (T0), 30 days (T30), and 90 days (T90) after its incremental increase to quantify platelet count, sP-sel (horse enzyme-linked immunosorbent assay) and PEVs (flow cytometry). Statistical analysis was performed using RM one-way analysis of variance with the Geisser–Greenhouse correction. Soluble P-selectin tended to increase at T30 compared with T0, while T90 levels returned to baseline values. Significantly higher circulating levels of PEVs CD61^+^/AnnV^+^ were observed at T30 and T90 compared to baseline confirming platelet hyperactivity. The detection and quantification of sP-sel and PEVs in equine racehorses during the training period appears to be a promising tool to study exercise-induced primary hemostatic changes and may provide an important marker for exercise selection.

## 1 Introduction

Physical exercise has an activating effect on the coagulation system and platelet function in healthy individuals, both in humans and racehorses. However, it is not clear whether this is beneficial or not. Interestingly, acute exercise induces effects that are different from those induced by long-term exercise training, and these effects are different in trained and untrained subjects, demonstrating that the hemostatic response depends on the type of exercise and training status (1–5).

Changes in hemostatic markers induced by long-term training in Thoroughbred horses have been reported (3, 6–10) showing contrasting results of hyper-or hypocoagulable response, probably due to differences in training schedules, breeds of horses, performance status, and methods used for analysis.

Recently, for the first time, we studied the effect of the first period of long-term incremental exercise training on the hemostatic system in untrained Thoroughbreds showing changes in platelet count, primary hemostasis (*in vitro* evaluation of platelet function assessed by platelet aggregation and PFA100^®^) and secondary hemostasis (Prothrombin Time, Partial Thromboplastin Time, Thrombin Clotting Time, Fibrinogen, thrombin-antithrombin complex-TAT). In particular, we observed thrombocytosis, an initial hypercoagulable state and platelet hyperreactivity at the initiation of training followed by downregulation of the hemostatic potential at the end of the training period (3). It is worth mentioning that alterations in platelet function appear to be implicated in exercise-induced pulmonary hemorrhage and hemorrhagic syndromes (EIPH) in equine athletes (11, 12). Platelets from horses affected by EIPH tend to be predisposed to thrombus formation and subsequent microvascular obstruction (11) and appear to be significantly less responsive to platelet agonists (12). These few data points suggest that further research on this topic, focusing on the effect of exercise on *in vivo* platelet function, is highly warranted.

In human studies, soluble P-selectin (sP-sel) and platelet-derived extracellular vesicles (PEVs) have gained increasing importance in recent years as reliable markers of *in vivo* platelet activation both in health and disease (13, 14).

P-selectin is a cell adhesion molecule that is stored in the alpha granules of platelets and in the Weibel-Palade bodies of endothelial cells and is expressed on their surface upon activation. A soluble form of P-selectin, derived from both platelet and endothelial sources, can be found in plasma as a circulating protein and it is considered not only a marker of platelet activation but also as direct inducer of pro-coagulant activity associated with vascular and thrombotic diseases in humans (15, 16).

PEVs are 0.1–1 μm vesicles shed from the plasma membrane of platelets upon activation with physiological agonists such as thrombin or collagen or in response to high-shear stress (17). In addition to their procoagulant function, several studies suggest a role for PEVs in inflammatory processes (18).

Both of these biomarkers are elevated in humans with cardiac and pulmonary diseases, cardiovascular disorders, sepsis, and diabetes mellitus (1, 19–21), and their dynamic changes and possible function induced by exercise have received much attention. It is well established that in untrained humans they increase after transient physical exercise, driven by vessels’ exposure to high-share stress, whereas long-term training induces a decrease in their resting levels due to an improved ability to adapt to changes in hemodynamics and cellular function during exercise (1, 22, 23).

To date, PEVs have been assessed in horses in a single study of resting animals (24). Information about the measurement of platelet-specific molecules released from platelet granules in equine samples is also limited (25).

The present study was designed to assess whether circulating levels of sP-sel and PEVs may be useful markers to explore *in vivo* platelet function in never-trained Thoroughbreds evaluating their dynamic changes during the first 4 months of incremental long-term training with the aim of improving knowledge on the fitness and health status of horses and possibly guiding the adjustment of training schedules.

## 2 Materials and methods

### 2.1 Animals

Animals included in the study were those reported in our previous studies (3, 10). A total of 29 clinically healthy (no history of hemostatic abnormalities; heart examination, thorax auscultation, rectal temperature, routine hematology, serum biochemistry, and hemostatic analyses were within reference limits) Thoroughbred racehorses (17 males and 12 females, 2 years of age) were enrolled. No pharmacological treatment was administered before or during the study.

Horses were housed in an AALAC-approved facility in accordance with protocols prescribed by the Institutional Animal Care and Use Committee. All horses were managed with individual housing, a natural photoperiod and natural indoor temperature, and the same feeding schedule. These animals were never trained for flat racing (canter and gallop) prior to entering this study. Each horse followed an individualized feeding program designed by a nutritionist and training team to maximize health conditions, performance, and well-being. Horses were fed three times a day (8:00 a.m., 12:00 noon, and 5:00 p.m.) with hay supplemented with green grass and mixed cereal concentrate. Water was available *ad libitum.*

All horses were trained according to the same training schedule (Table 1). Training was performed at the same time for each horse from Monday to Saturday with 1 day of rest (Sunday), for a 90-day period. Clinical evaluations and blood samples were collected at rest, before training, on the day of initiation of the training program, which included canter and gallop (T0), 30 days (T30), and 90 days (T90) after the incremental increase in the training program to assess whether or not the animals had adapted to the exercises. Each animal competed in one or more races after the end of the experimental period. None of the horses exhibited poor performance during the study period or during the races.

**Table 1:** Standard daily training program completed by each horse involved in the study.

April (T0) Start of training	May (T30) Incremental training	July (T90) Incremental training
15 min Walk	15 min Walk	15 min Walk
10 min Trot	10 min Trot	10 min Trot
6 min Canter	6 min Canter	6 min Canter
Every Tuesday	Every Tuesday	Every Tuesday
1 min Gallop	2 min Gallop	4 min Gallop

Speeds: Walk: 100 m/min, Trot: 200 m/min, Canter: 350 m/min, and Gallop: 1000 m/min (min: minutes).

### 2.2 Sample collection

Blood sampling was performed over a 4-month period from April to July, at 6:30 a.m., at rest, before training and feeding. At T90, the sample collection was done before the race. April (T0) was the sample before the first month of training simulating competition (gallop). From April to July, training was incremental. Blood samples were collected by venipuncture of the jugular vein, paying attention to avoid unnecessary manipulation of the sampling site, which could result in the activation of coagulation. Blood samples were collected in Vacutainer tubes (Terumo Corporation, BD brand; Tokyo, Japan) containing K3-ethylenediamine tetra acetic acid-EDTA and 3.2% sodium citrate. The first few milliliters were discarded to avoid the potential artifacts generated by the contact phase activation. Moreover, to avoid cross-contamination of additives between tubes, blood was drawn and collected in tubes in the following order: first sodium citrate and then K3-EDTA tubes. All the tubes were immediately transported to the local hospital at room temperature to limit cold-induced activation of platelets and were processed within 2 h of collection. K3-EDTA tubes were used to evaluate the platelet counts using an automated hematology analyzer (Sysmex-XT1800iV; Sysmex, Kobe, Japan) validated for horses and equipped with multispecies software. Sodium citrate tubes were centrifuged at 4,000 g for 10 min to obtain platelet-poor plasma (PPP). PPP was then divided into small aliquots in pre-labeled tubes to minimize freeze–thaw cycles and frozen at −80°C until assayed.

For the detection and quantification of PEVs, 1 mL of PPP was further centrifuged for 2 min at 13,000 g and the resulting supernatant was stored at −80°C (24, 26, 27).

### 2.3 Soluble P-selectin

Soluble P-Selectin was measured in PPP from citrated blood using a horse enzyme-linked immunosorbent assay (ELISA) (MyBioSource). The sensitivity was 1 ng/mL while the intra- and inter-assay CV (%) was less than 15%.

### 2.4 Platelet-derived extracellular vesicles

In total, 50 μL of PFP were incubated with 2.5 μL anti-GPIIb/IIIa antibody (CD61FITC, MyBioSource, clone JM2E5) plus 2 μL AnnexinV-PE (PEVs CD61^+^/AnnV^+^) for 30 min at room temperature in the dark (24). The reaction was stopped with 500 μL of calcium-rich binding buffer and a known volume (identical to that of the sample) and the concentration of Flow Count Fluorospheres (Beckman Coulter) was added prior to analysis (26, 27). The morphological gate for extracellular vesicles was set using Megamix (BioCytex, Marseille, France), a mix of fluorescent beads of various diameters covering the size range of microvesicles (0.5 and 0.9 μm) and platelets (0.9 and 3 μm), according to the recommendations of international guidelines (28). After analysis on the flow cytometer, the absolute count was automatically calculated by the instrument software using the Assayed Concentration (in our experiments, 998/μl), using the following formula: (total number of cells counted/total number of flow counts) × flow-count assayed concentration. More details are provided in the Supplementary material.

### 2.5 Statistical methods

All results are expressed as mean ± standard error (SEM). Differences between time points were analyzed by the RM one-way analysis of variance with Geisser–Greenhouse correction, and the mixed-effects model was applied and adjusted by Tukey’s multiple comparison test, with individual variances computed for each comparison; initial values were based on GLM. *p* < 0.05 was considered statistically significant.

## 3 Results

### 3.1 Effect of training on platelet counts

No significant effect of training on platelet counts was observed at 30 and 90 days compared with basal (T0). The mean values were within the reference ranges (Supplementary Table S1).

### 3.2 Effect of training on sP-selectin

**Figure 1:**
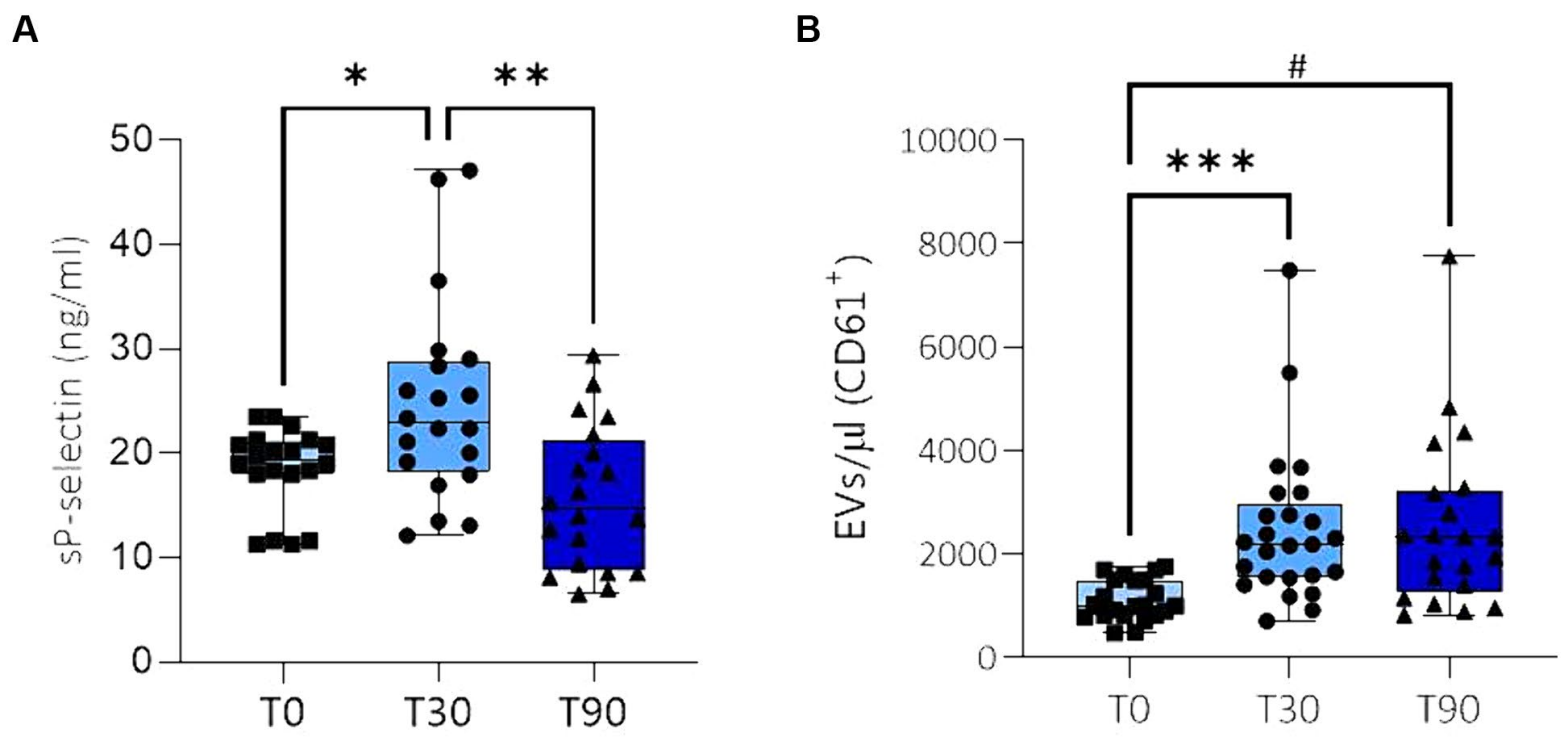
Levels of sP-sel **(A)** and PEVs CD61^+^/AnnV^+^ **(B)** in plasma collected on the day of the beginning of the training program, which included canter and gallop (T0) and 30 days (T30) and 90 days (T90) after the incremental increase of the training program. *p = 0.0143; **p = 0.0020; ***p = 0.0006; # = 0.0011.

Soluble P-selectin increased after 30 days (T30) of the training program compared to baseline (T0), whereas after 90 days (T90) of the training program, levels returned to baseline (Figure 1A; Supplementary Table S1).

### 3.3 Effect of training on PEVs

Significantly higher circulating levels of PEVs CD61^+^/AnnV^+^ were observed at T30 compared to baseline confirming platelet hyperreactivity. This increase was maintained also after 90 days of the training program compared to the baseline (Figure 1B; Supplementary Table S1).

## 4 Discussion

The present study reports for the first time the effect of long-term training on circulating levels of *in vivo* platelet activation markers, sP-sel, and PEVs CD61^+^/AnnV^+^, in healthy, untrained racehorses. Our results show that sP-sel and PEVs CD61^+^/AnnV^+^ levels increased 1 month after the start of training, although at the border of significance for sP-Sel, confirming a condition of platelet hyperreactivity and *in vivo* platelet activation demonstrating the exercise-induced changes in hemostasis as supported by previous results in humans (29).

It is well known that exercise intensity, duration, type, and individual training status are important factors involved in exercise-induced changes in platelet *in vivo* and platelet reactivity function; in fact, *in vivo*, platelet activation appears to be suppressed in the course of moderate exercise, whereas it is increased during strenuous exercise (1, 30, 31).

P-selectin is an adhesion receptor molecule found in platelet α-granules and in the Weibel-Palade bodies of endothelial cells. A soluble form of P-selectin can be found in plasma as a circulating protein that is shed from endothelial cells and the platelet surface membrane after activation, although platelets are currently considered to be the major source of circulating soluble P-selectin in healthy individuals (32). Its biological role is unknown, but its levels are commonly used as a reliable marker of *in vivo* platelet activation, capable of predicting a pro-thrombotic state in both humans and various animal species (15, 33). It appears to be an agonist of the coagulation process mediating platelet–platelet and platelet-leukocyte interactions and favoring irreversible platelet aggregation upon thrombin stimulation (34).

A significant increase in sP-sel has been found following strenuous exercise in humans, although its release mechanism has not been well characterized (2).

PEVs are a heterogeneous population of small circular platelet membrane fragments generated during platelet activation by various stimuli, senescence, and apoptosis. They retain membrane antigens and cytoplasmic components of their cellular origin and play an essential role in coagulation. Elevated concentrations of circulating PEVs have been reported in thrombotic conditions in humans and are considered an independent predictor of poor outcomes.

Moreover, in humans, the level of PEVs offers insight into the state of circulating platelets during exercise and is considered a useful marker for monitoring physical training activities. The level of PEVs appears to be related to training intensity, with little change during moderate exercise and a high increase during high-intensity exercise (29, 35). Blood levels of EVs change immediately and remain high for up to 3 h after a single bout of exercise (1).

Interestingly, in our study, circulating EV levels increased significantly 1 month after the beginning of training and remained high until the end of the training period while sP-sel increased after 30 days of training and tended to return to basal levels at the end of the training period.

The circulating levels of soluble adhesion molecules are likely to be determined not only by their synthesis and shedding but also by their clearance. Little is known about the clearance of soluble selectins such as P-selectin and we can assume that incremental training could increase the clearance of these soluble molecules. Similarly, our finding that PEV levels are persistently elevated after long-term training may reflect that, although the detailed clearance mechanism of EVs in circulation is not clear, the release and clearance of EVs may coexist during exercise.

Flow cytometry is considered the gold standard method for the study of EVs, allowing the accurate identification and enumeration of EV subpopulations based on the use of distinct monoclonal antibodies that detect cell-specific surface proteins in both humans and horses (1, 36, 37). In this study, we applied a standardized flow cytometry protocol for the identification and enumeration of EVs in equine plasma using an anti-CD61 antibody, as previously reported (24). Moreover, we adapted our protocol from those standardized for humans, which are able to minimize preanalytical influences on PEV counts (38) with the advantage of good accuracy and ease of application.

Platelets are affected by a multitude of physical and chemical factors including sampling collection, handling, processing, and artificial *in vitro* activation, which may limit the reliability of results. In our study, precautions were taken to minimize *ex vivo* and *in vitro* platelet activation according to international guidelines (28).

The present results confirm our previous observations showing an initial hypercoagulability state and increased *in vitro* platelet reactivity induced by incremental training in thoroughbred horses, which reached maximum levels at 30 days and then tended to return to baseline at the end of the exercise period (3, 10).

The initial hypercoagulable state places racehorses at risk for thrombosis with its clinical sequelae of microthrombotic events, ischemic tissue injury, and organ dysfunction (12, 39–42). Since platelets play a crucial role in the process of thrombus formation and have a pro-inflammatory effect, this vicious cycle between inflammation and coagulation may contribute to the pathogenesis of different common pathologies affecting sport horses such as laminitis, acute renal failure, and EIPH. For this reason, early identification of subclinical alterations in hemostasis in training horses may be of help in the management of equine athletes (11, 43).

This study had some limitations, such as the limited number of animals and the lack of a control group that did not enter training; however, we sampled a group of horses that had never been trained before to have control over untrained subjects. Also, to reduce environmental effects, we waited 30 days before housing the animals in the same training center, which standardized management and diet. Moreover, the lack of a CD62P marker for the identification of MVs does not allow us to discriminate between circulating microparticles derived from platelets following activation and those continuously derived from megakaryocytes although we used a commonly used double staining approach (CD61^+^/AnnV^+^) for the identification of MVs (38).

Larger prospective studies testing the effect of regular exercise training on platelet function in healthy horses during training are warranted.

## 5 Conclusion

The detection and quantification of soluble P-selectin and PEVs in equine racehorses during the training period appears to be a promising tool to study exercise-induced changes in primary hemostasis and further studies are warranted to better establish reference ranges. The changes in sP-sel and MVs in healthy Thoroughbreds during their first long-term training may provide an important surrogate marker for exercise selection in racehorses. Future studies with larger numbers of racehorses are needed to reassess the possible relationship between *in vivo* platelet activation, intensity of training, and training status.

## Data availability statement

The original contributions presented in the study are included in the article/Supplementary material, further inquiries can be directed to the corresponding author.

## Ethics statement

Ethical approval was not required for the studies involving animals in accordance with the local legislation and institutional requirements because The study was performed on material collected during clinical procedures. Blood sampling was approved by the Italian Horse Racing Board and performed by the authorized veterinary practitioner during routine controls to assess the health of the animals in the course of the training season. Prior to sample collection, written owner consent was obtained for all animals included in the study. Animal care procedures were compliant with the European recommendations (Directive 2010/63/EU) for protecting animals used for scientific purposes. Written informed consent was obtained from the owners for the participation of their animals in this study.

## Author contributions

AMi: Writing – review & editing, Writing – original draft, Visualization, Validation, Supervision, Software, Resources, Project administration, Methodology, Investigation, Funding acquisition, Formal Analysis, Data curation, Conceptualization. EF: Writing – original draft, Visualization, Validation, Software, Methodology, Formal Analysis, Data curation, Conceptualization. AMe: Writing – review & editing, Visualization, Validation, Formal Analysis, Data curation. SB: Writing – review & editing, Validation. FR: Writing – review & editing, Validation, Funding acquisition. PG: Writing – review & editing, Validation, Supervision, Methodology, Funding acquisition, Conceptualization. MA: Writing – review & editing, Visualization.
