# Supplementary material for: Biomarkers of in vivo platelet activation in thoroughbreds during their first long-term training
Source: Front Vet Sci. 2024 May 20;11:1395423. doi: 10.3389/fvets.2024.1395423 (PMC11145980; doi:10.3389/fvets.2024.1395423)
Supplement: Supplementary file 1 [file Data_Sheet_1.PDF]

Suppl. Figure 1. Gating strategy and representative scatter dot plots of flow cytometric detection and quantification of PEVs.

A) Instrument set-up. Megamix beads distribution in the FSC x FITC-A dot plot (*i*). Identification of PEVs region boundaries (MP gate, 0.3-0.9µM) (*ii*).

B) PEVs measurement in equine plasma samples. PEVs distribution in the MP gate (*i*). Detection of Annexin V-PE and CD61-FITC stained PEV (*ii*) and their quantification by using Flow-Count Fluorospheres, counted in a two-parameter (dual color fluorescence) density plot (*iii*) and also in a PC5 fluorescence over time plot (to evaluate also their homogeneity) (*iv*).

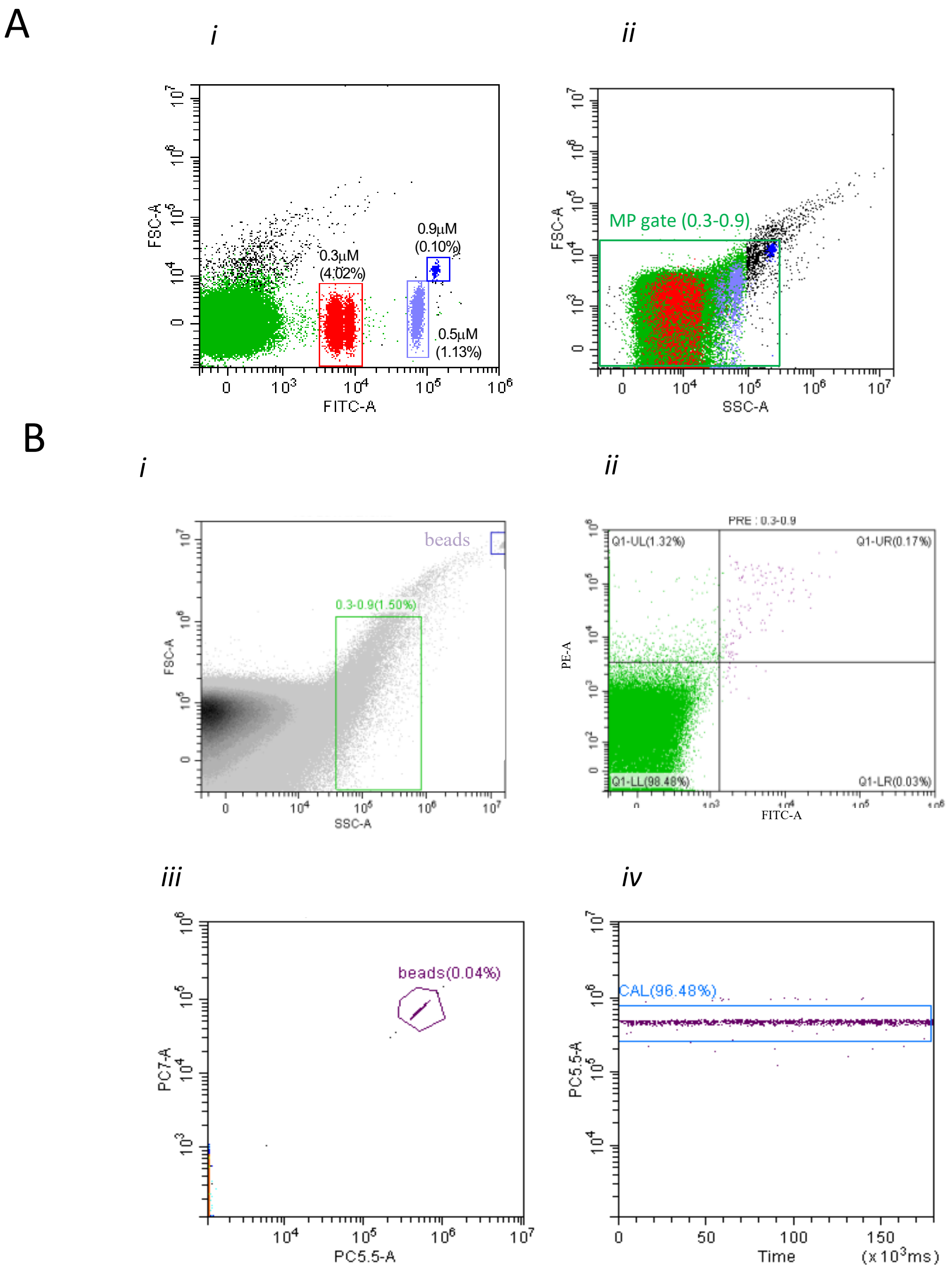

Supplementary Table 1. Platelet count (PLT), soluble P-selectin (sP-sel) and platelet-derived extracellular vesicles (PEVs $CD61^+/AnnV^+$) at baseline and during the training program. Data are reported as mean±SEM. Significant differences are expressed with * $p<0.05$ vs T90, $^{**}p<0.05$ vs T0.

Parameters	T0	T30	T90
**PLT (×10^9/L)**	151±35	166±52	149±42
**sP-sel (ng/ml)**	18.7±0.8	24.8±1.9*	15.7±1.5
**PEVs $CD61^+/AnnV^+$ (n/μl)**	1438±56	1880±84**	1729±57**
